# ST2/IL-33 signaling promotes malignant development of experimental squamous cell carcinoma by decreasing NK cells cytotoxicity and modulating the intratumoral cell infiltrate

**DOI:** 10.18632/oncotarget.25768

**Published:** 2018-07-20

**Authors:** Nádia Ghinelli Amôr, Carine Ervolino de Oliveira, Thaís Helena Gasparoto, Vanessa Garcia Vilas Boas, Graziela Perri, Ramon Kaneno, Vanessa Soares Lara, Gustavo Pompermaier Garlet, João Santana da Silva, Gislâine A. Martins, Cory Hogaboam, Karen A. Cavassani, Ana Paula Campanelli

**Affiliations:** ^1^ Department of Biological Sciences, Bauru School of Dentistry, University of São Paulo, Al. Dr. Octávio Pinheiro Brisolla, Bauru, SP, 17012-901, Brazil; ^2^ Department of Microbiology and Immunology, Institute of Biosciences of Botucatu, São Paulo State University, R. Prof. Dr. Antônio Celso Wagner Zanin, Botucatu, SP, 18618-689, Brazil; ^3^ Department of Stomatology – Oral Pathology, Bauru School of Dentistry, University of São Paulo, Al. Dr. Octávio Pinheiro Brisolla, Bauru, SP, 17012-901, Brazil; ^4^ Department of Biochemistry and Immunology, School of Medicine of Ribeirão Preto, University of São Paulo, Ribeirão Preto, SP, 14049-900, Brazil; ^5^ Department of Biomedical Sciences (Research Division of Immunology) and Medicine, F. Widjaja Foundation Inflammatory Bowel and Immunobiology Research Institute, Cedars-Sinai Medical Center, Los Angeles, CA, 90048, USA; ^6^ Department of Medicine, Division of Pulmonary and Critical Care Medicine, Cedars-Sinai Medical Center, Los Angeles, CA, 90048, USA; ^7^ Samuel Oschin Comprehensive Cancer Institute, Cedars-Sinai Medical Center, Los Angeles, CA, 90048, USA

**Keywords:** squamous cell carcinoma, IL-33, ST2, chemical carcinogenesis, immune modulation

## Abstract

Squamous cell carcinoma (SCC) is the second most common form of skin cancer and the mechanism(s) involved in the progression of this tumor are unknown. Increases in the expression of IL-33/ST2 axis components have been demonstrated to contribute to neoplastic transformation in several tumor models and interleukin-33 is correlated with poor prognosis of patients with squamous cell carcinoma of the tongue. Based on these observations, we sought to determine the role of the IL-33/ST2 pathway during the development of SCC. Our findings show that ST2-deficiency led to a marked decrease in the severity of skin lesions, suggesting that ST2 signaling contributed to tumor development. An analysis of tumor lesions in wild-type and ST2KO mice revealed that a lack of ST2 was associated with specific and significant reductions in the numbers of CD4^+^ T cells, CD8^+^ T cells, dendritic cells, and macrophages. In addition, NK cells that were isolated from ST2KO mice exhibited higher cytotoxic activity than cells isolated from wild-type mice. Notably, ST2 deficiency resulted in lower IFN-γ, TNF-α, IL-10, and IL-17 production in tumor samples. Our findings indicate that the IL-33/ST2 pathway contributes to the development of SCC by affecting leukocyte migration to tumor microenvironment and impairing NK cytotoxic activity.

## INTRODUCTION

Squamous cell carcinoma (SCC) is the second most common human skin cancer [1]. It occurs primarily in Caucasians, especially those living in tropical regions. The rate of cutaneous SCC dissemination can reach 16% [2], but the mechanisms underlying SCC progression are poorly understood. Studies of the pathogenic mechanisms involved in SCC development have reported that the tumor microenvironment comprises a complex system of many cell types, including endothelial cells; fibroblasts; neutrophils and other granulocytes; mast cells; T, B, and natural killer lymphocytes; macrophages and dendritic cells, which are known to influence the initiation and progression of carcinomas [3, 4]. In a model of experimental SCC, it was demonstrated that carcinoma-associated T cells and IFN-γ were key effectors of the anti-tumor immune response [5]. Additionally, different cytokines have been shown to either promote or inhibit tumor development and progression, regardless of their source [6, 7]. The most recent cytokine to be associated with tumor progression is interleukin (IL)-33.

IL-33 is a member of the IL-1 family of cytokines and was identified as a ligand for the T1/ST2 receptor (ST2; also called ST2L, IL-33R, or IL-1RL1) [8]. The binding of IL-33 to its receptor, ST2, induces the MyD88-dependent activation of NF-κB and MAPK [9]. ST2 is expressed on many immune cells [10], and the IL-33/ST2 signaling pathway augments IL-5 and IL-13 production by Th2 cells [8]. Now, evidence is accumulating that IL-33 also potently stimulates group 2 innate lymphoid cells (ILC2s), regulatory T (Treg) cells, Th1 cells, CD8^+^ T cells and natural killer (NK) cells [reviewed by [11]. While the cytokine IL-33 appears to have pleiotropic functions in diverse types of inflammatory diseases, including inflammatory bowel disease [12], asthma/allergic diseases [13], and atherosclerosis [14], its function in tumorigenesis remains controversial. IL-33 is described as a crucial mediator of carcinogenesis in inflammation-associated pancreatic cancer [15]. Similarly, in human head and neck SCC, IL-33 has been associated with tumor cell invasion and metastasis [16, 17]. However, in Lewis lung carcinoma, the transgenic expression of IL-33 inhibited tumor growth and metastasis in mice [18]. Therefore, the role of IL-33 and its contribution to carcinogenesis remains to be clarified.

Based on these observations, we sought to determine the role of the IL-33/ST2 pathway during the development of SCC. We show that the IL-33/ST2 interaction is necessary for SCC progression, that it is involved in a mechanism during the recruitment of CD4^+^ T lymphocytes, dendritic cells, and macrophages in the tumor microenvironment, and that it promotes the production of IFN-γ, TNF-α, IL-10, and IL-17. In addition, a lack of ST2 was associated with increased NK cells cytotoxicity. Based on our data, we suggest that IL-33/ST2 signaling modulate immune cells infiltration in the tumor microenvironment and NK cells activity favoring tumor development. Inducing a blockade of IL-33/ST2 signaling may represent an attractive approach to improving current therapies to combat cutaneous SCC.

## RESULTS

### IL-33/ST2 signaling favors SCC development

**Figure 1:**
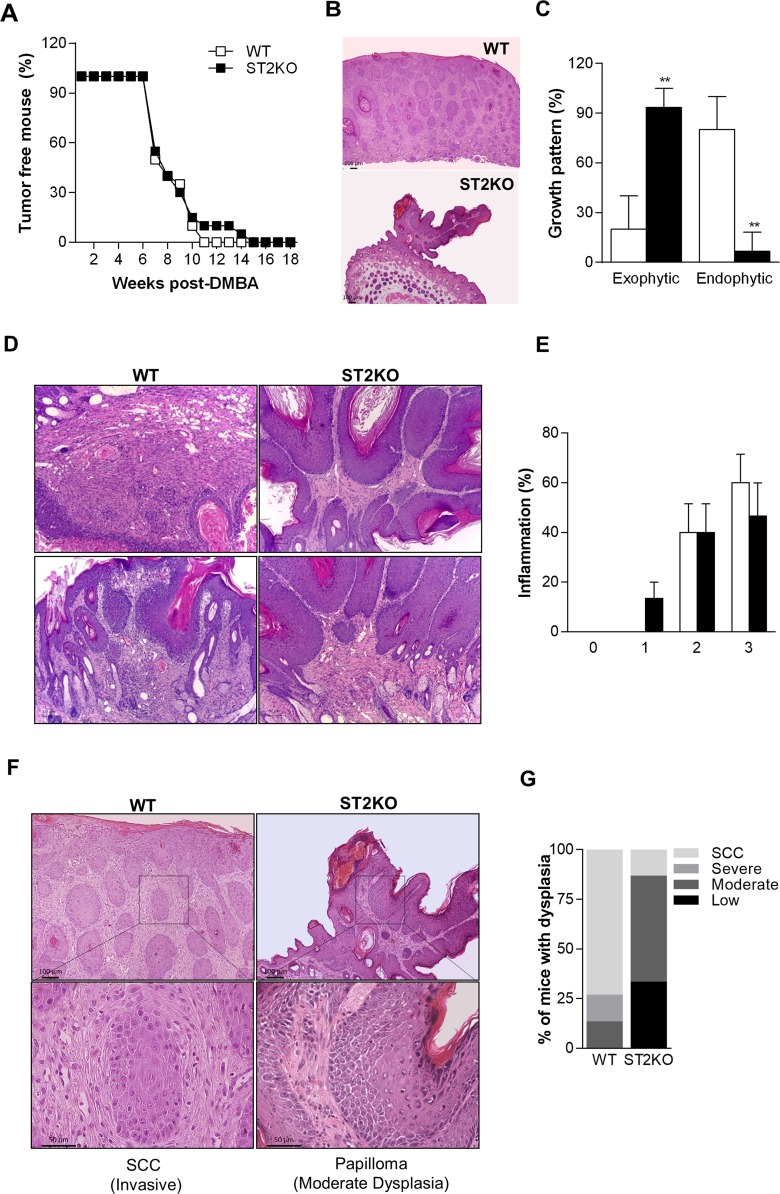
ST2/IL-33 signaling favors SCC development **(A)** Graphic showing the frequencies of tumor-free wild-type (WT, empty squares) and ST2KO mice (full squares). The results are expressed as the percentage of mice that were free of skin tumors (n = 15 per group). **(B)** Representative pictures of lesion growth patterns (40x). **(C)** Graphic showing the frequencies of exophytic and endophytic tumors. **(D)** Representative pictures of tumor inflammatory infiltration (100x). Scale bars, 50μM **(E)** Graphic showing the inflammation score in wild type and ST2KO mice. **(F)** Representative pictures are shown of H&E-stained skin sections that were obtained from wild type and ST2KO mice at 18 weeks after SCC induction (100x (upper) and 400x (lower) magnification). Scale bars, 50μM (lower), 100μM (upper). **(G)** H&E stained sections were scored to grade their level of dysplasia. Scale bars, 50μM. The data represent the mean ± SEM from 3 independent experiments. ^**^P < 0.01.

To explore the role of IL-33 during SCC development, ST2-deficient (ST2KO) and wild type (WT) mice were submitted to a multistage tumorigenesis protocol [19]. The average first appearance of tumors was recorded during all experiment (Figure 1A). Both WT and ST2KO mice had developed lesions at 7 weeks post-DMBA treatment (Figure 1A). Interestingly, 100% of the WT mice had developed lesions at 11 weeks after SCC induction, while lesions were detected in 100% of the ST2KO mice at 15 weeks post-DMBA exposure (Figure 1A). A histological analysis revealed that the tumors in the wild type mice presented a mostly endophytic tumor growth pattern (lesion that grows inward from an epithelial surface), whereas the tumors in the ST2KO mice presented a primarily exophytic pattern (lesion that grows outward from an epithelial surface) (Figure 1B and 1C). H&E-stained skin sections were scored blindly to evaluate inflammation and hyperplasia. Although no significant difference was observed in inflammation in the histological analysis, we found that the ST2KO mice tumors presented less inflammatory infiltration than the WT mice (Figure 1D and 1E). The semi-quantitative scores for hyperplasia showed that 90% of the ST2KO mice displayed low to moderate-grade dysplasia, while only 10% were classified as SCC *in situ* (Figure 1F and 1G), 30% of the WT mice developed moderate to severe dysplasia, and 70% of these were classified as SCC (Figure 1F and 1G). Thus, our results indicate that ST2-deficient mice are less susceptible to developing SCC in the skin.

### ST2 deficiency was associated with reduced numbers of CD4^+^ and CD8^+^ T cells, dendritic cells, and macrophages in the tumor microenvironment

**Figure 2:**
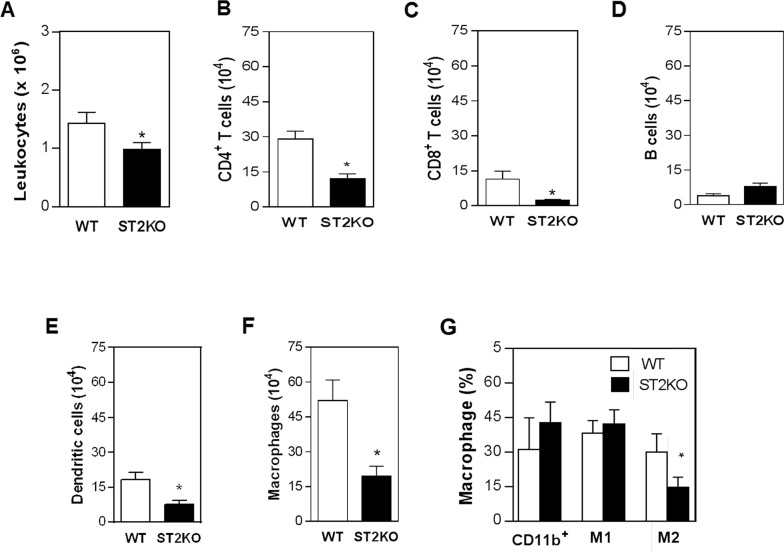
ST2 deficiency is associated with reduced immune cell infiltration in tumors The frequencies of macrophages (F4/80^+^), B (CD19^+^) and T cells (CD4^+^ and CD8^+^), and dendritic cells (CD11c^+^) were determined using immunostaining and FACS analysis in tumor lesions that were harvested from mice on day 126. The bars show **(A)** the absolute number of leukocytes and the number of **(B)** CD4^+^ T lymphocytes, **(C)** CD8^+^ T lymphocytes, **(D)** B lymphocytes, **(E)** dendritic cells, **(F)** and macrophages present in the tumor lesions. **(G)** Frequencies of CD11b^+^ (myeloid cells), CD119^+^ (M1) and CD124^+^ (M2) cells in the tumor microenvironment. The data are representative of three experiments (n = 5 per group). ^*^P < 0.05.

To determine how IL-33/ST2 might promote SCC development, we next characterized the immune cell infiltrate of the tumor microenvironment. The absolute number of leukocytes in the tumor lesions from ST2KO mice was significantly lower than WT mice (Figure 2A). Moreover, a significantly lower number of dendritic cells, macrophages, CD8^+^ T and CD4^+^ T cells were contained in the tumors obtained from ST2KO mice than in the tumors obtained from WT mice (Supplementary Figure 1, and Figure 2B-2C and 2E-2F). Numbers of B-lymphocytes were similar between these two groups (Figure 2D). Analysis of macrophages phenotypes shows no difference in M1 macrophages frequency among the groups (Figure 2G). However, ST2KO mice display a significant decrease in the frequency of M2 macrophages compared to WT mice (Figure 2G). Thus, our results show that the IL-33/ST2 interaction is required for SCC progression and that it is involved in a mechanism that contributes to the recruitment of CD4^+^ T lymphocytes, dendritic cells, and macrophages, predominantly the M2 phenotype, to the tumor microenvironment.

### The IL-33/ST2 axis affects the cytotoxic activity of NK cells

**Figure 3:**
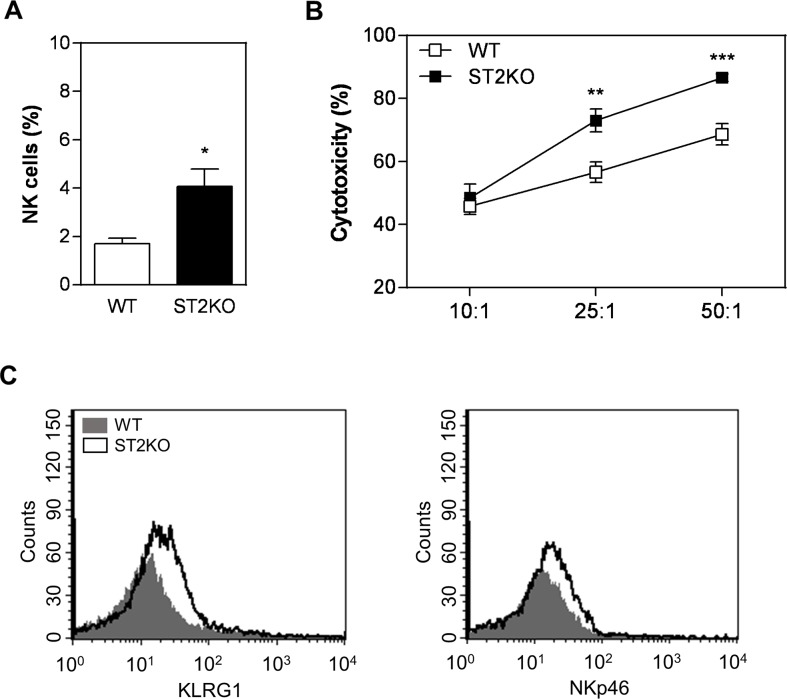
A lack of ST2 is associated with increased NK cytotoxicity **(A)** Flow cytometry was used to quantify the proportions of NK cells (CD11b^+^CD27^+^) in tumor samples obtained from wild type and ST2KO mice. **(B)** Flow cytometry analysis of splenic NK cell activity against YAC-1 cells and **(C)** KLRG1 and NKp46 expression: WT, grey histogram; and ST2KO, empty histogram. The data shown are representative of the mean ± SEM from at least three independent experiments (n = 5 per group). ^*^P < 0.05, ^**^P < 0.01; ^***^P < 0.001.

Because recent studies have shown that IL-33 enhances the function of effector NK cells [20, 21], we next considered whether ST2 deficiency affects NK cytotoxicity in our SCC tumor model. To investigate this possibility, we first analyzed the proportion of NK cells in the tumor microenvironment in WT and ST2KO mice. We found that ST2-deficiency resulted in an increase in the percentage of NK cells in the tumor microenvironment (Figure 3A). These data indicated that signals downstream of ST2/IL33 might interfere with NK cell recruitment and/or survival *in vivo*. Furthermore, ST2-deficient NK cells exhibited higher cytotoxic activity than WT cells *in vitro* (Figure 3B). Finally, we analyzed the expression of two major molecules associated with NK cell activity, and our results show that lack of ST2 increases KLRG1 and NKp46 expression (Figure 3C). Thus, the IL-33/ST2 interaction is associated with decreased frequencies of NK cells in the tumor environment and decreased cytotoxic activity, indicating that IL33/ST2 plays a suppressive role in NK cell functions during tumor development.

### IL-33/ST2 contributes to SCC development by increasing the production of inflammatory cytokines

**Figure 4:**
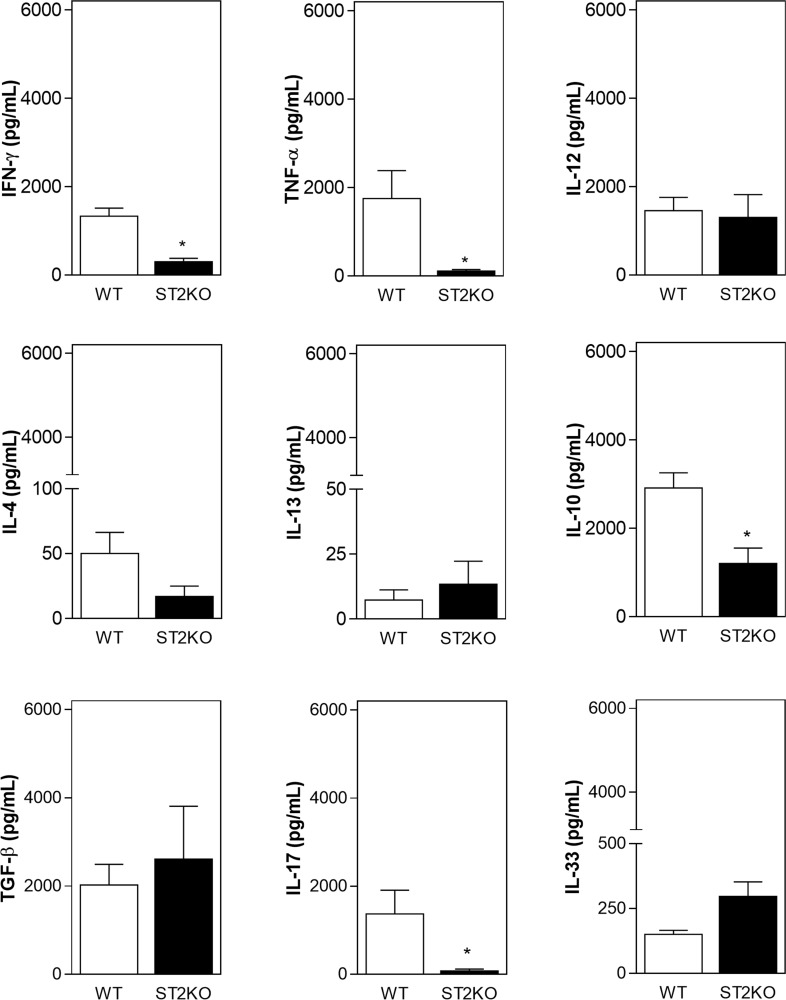
A lack of ST2 is associated with a decrease in the expression of pro-inflammatory cytokines IFN-γ, TNF-α, IL-12p70, IL-4, IL-10, IL-13, TGF-β, IL-17, and IL-33 levels were measured in supernatants recovered during tumor processing using ELISA. The data are shown as the mean ± SEM and are representative of at least three independent experiments (n = 5 per group). ^*^P < 0.05.

Given the inflammatory infiltrate patterns that we observed in the tumor microenvironment, we next sought to determine whether ST2 deficiency regulates tumor development in a cytokine-dependent manner. Tumor samples obtained from ST2KO mice presented lower levels of IFN-γ, TNF-α, IL-10, and IL-17 (Figure 4), while the production of IL-12, IL-4, IL-13 and TGF-β was similar between tumor skin samples obtained from wild type and ST2KO mice (Figure 4). IL-33 levels were also slightly but not significantly higher in the samples obtained from ST2-deficient mice than in those obtained from WT mice. Collectively, these data indicate that cytokine production is affected by the IL33/ST2 axis during the anti-tumor immune response.

## DISCUSSION

Evidence indicates that IL-33 is a possible inducer of and prognostic marker for cancer development [22]. However, its precise function during SCC development has not been defined. In this study, we found that ST2-deficient mice are less susceptible than wild type mice to SCC development in the skin. This finding adds new insight into the role of IL-33 in tumorigenesis. It was previously shown that the IL-33/ST2 axis is key to the process of human SCC progression [16]. The data presented in this report further extend these findings by showing that ST2-deficiency leads to decreased susceptibility to tumor development, which is associated with moderate dysplasia, exophytic growth pattern and low frequency of malignant conversion. In our model, less than 10% papillomas progress to SCC in ST2-deficient mice while up to 70% of papillomas that develop in WT mice may convert to invasive SCC.

The development of SCC is a multistep process that is characterized by the unbalanced production of pro- and anti-inflammatory cytokines and infiltration by macrophages and lymphocytes into the tumor microenvironment [3, 4]. Our study shows that the absence of IL-33 signaling affects the recruitment of CD4^+^ T cells, dendritic cells, macrophages, and NK cells to the tumor microenvironment. These results imply that IL-33 regulates crucial aspects of immune cells trafficking during SCC progression by direct or indirectly affecting leukocyte migration to inflamed tissues. We might speculate, then, that absence of ST2 influenced tumor microenvironment and lead a reduction of chemokines. A recent study showed that signaling via IL-33 induced eosinophils to migrate in response to an increase in the production and activation of ILC2 [23]. In addition, in a mouse model of breast cancer, the administration of exogenous IL-33 resulted in the production of a mixed regulatory/Th2 immune infiltrate that promoted tumor growth [24]. Although the exact mechanism by which IL-33 increases the migration of these cells to the tumor microenvironment remains to be determined, it is likely that the IL-33 that is produced in the tumor environment acts locally on resident and inflammatory cells to induce the secretion of chemokines and the subsequent recruitment of immune cells to the tumor microenvironment.

Studies have shown that the presence of infiltrating CD4^+^ T cells and macrophages in tumors is associated with neoplastic progression [25, 26]. Our results show that higher frequency of CD4^+^ T cells are associated with SCC development. Indeed, this association between an increased number of CD4^+^ cells and SCC development has previously been observed in mice and humans [25]. CD4^+^ T lymphocytes are crucial mediators of immune responses that can play opposing functions during tumor development, with their role depending on influences within the microenvironment. Tumorigenesis is commonly associated with the suppression of cell-mediated Th1 immunity and enhanced humoral immunity (Th2) [27]. In addition, it has been shown that the IL-33/ST2 interaction drives the polarization of naïve T cells towards a Th2 phenotype [28] and that blocking this pathway amplifies anti-tumor Th1-immune responses [24]. However, recent studies indicate that IL-33 can also support type 1 responses [29]. In our study, the frequencies of specific tumor-associated CD4^+^ T cell subsets, which can be distinguished by markers such as Th2, Th1 or Th17 cytokines, were not determined. But, the level of IL-10, a Th2 cytokine, was significantly higher in the tumor microenvironment of wild type mice that developed invasive SCC, and only relatively low concentrations of IL-4 and IL-13 were detected. IL-10 is an immunosuppressive and anti-inflammatory cytokine linked with inflammation-associated cancer [30]. IL-10 exerts a wide range of effects on resident and circulating immune cells, including inhibition of pro-inflammatory cytokines production, inhibition of Th1 polarization and decreasing inflammation [31, 32]. In addition, the overexpression of IL-10 in the tumor was associated with an increase in tumor cell proliferation, and immune evasion [33], which corroborates our findings indicating a possible correlation between overexpression of IL-10 and tumor development. In our experimental conditions, we also observed that the TNF-α levels were significantly higher in the tumors of WT mice, which is consistent with the ability of TNF-α to induce inflammation and subsequent tumor development [34]. TNF-α is an important mediator of inflammation and has been correlated to several steps involved in carcinogenesis, for example, cellular transformation, survival, proliferation, invasion, angiogenesis, and metastasis [35, 36]. A lack of TNF-α has been correlated with resistance to skin carcinogenesis [37], which corroborates our findings indicating the presence of a direct relationship between high TNF-α levels and tumor development.

The present study showed that Th1-signature cytokine IFN-γ was significantly lower in the tumors of ST2-deficient mice that not developed invasive SCC. Regardless of the general knowledge that IL-33 is is primarily associated with Th2-type cytokine profiles, new evidence demonstrated that IL-33 also regulates pro-inflammatory cytokines, including the ones involved with Th1 immune responses [20]. In addition, *in vivo* blockage of ST2 significantly decreased the levels of IFN-γ and IL-17 suggesting that ST2/IL-33 axis also modulates Th1-related cytokines [38]. While IFN-γ is described to potentiating anti-tumor immunity by inducing CTL activation, and dendritic cell activity [39], a clear association with skin papillomas development is described [40]. Therefore, considering the chronic nature of tumor microenvironment and the reduced presence of T cells in ST2KO lesions, it would be possible to suggest that the pro- or anti-tumoral IFN-γ activity within the tumor microenvironment depends not only on local concentration but also on its expression during tumor development [41]. With regard for Th17 subsets, our results show that significantly higher levels of this cytokine were observed in the tumor microenvironment in wild type mice that developed invasive SCC, and these data provide insight into the mechanisms that mediate SCC development. Importantly, recent studies have demonstrated that IL-33 has the ability to directly stimulate mast cells and enhance the Th17 response during the pathogenesis of asthma [42]. Higher IL-17 levels were detected in the tumor microenvironment in wild type mice, which displayed invasive SCC development. Evidence has also indicated that IL-17 increases macrophage recruitment at the tumor site and indirectly promotes M2 macrophage differentiation in the tumor microenvironment [43]. These data suggest that IL-33 and IL-17 may act locally on resident and inflammatory cells to induce the secretion of chemokines and the subsequent recruitment of M2 macrophages to the tumor microenvironment, which would favor the development of invasive SCC. In fact, our results showing that invasive SCC development has associated with a high number of M2-type macrophages in the tumor microenvironment in wild type mice, and lack of ST2 significantly decrease the frequency of M2-type macrophages, and consequently reduce SCC progression. Pro-tumorigenic TAMs express molecules that are typical of an M2 phenotype [44], and IL-33 can drive M2 polarization [45]. Therefore, we suggest that in this model, the IL-33/ST2 pathway potentiated M2 phenotype polarization, which resulted in the development of more severe SCC than was observed in WT mice. The interplay between IL-33 and immune cells is potentially a mechanism that mediated susceptibility to SCC development in this model. Further analysis is required to support this hypothesis.

We have also shown that the deleterious effects induced by IL-33 during SCC progression are, at least in part, associated with a reduction in NK frequencies. A recent study indicates that due to their powerful cytotoxic activity NK cells can play important roles in the immune response against tumors even when present at low frequencies [46]. We have also detected that IL-33/ST2 signaling affects NK cytotoxic activity. These data are in agreement with the study showing that IL-33 can decrease NK cell activity [47]. In this study, deletion of ST2 in BALB/c mice bearing mammary carcinoma attenuated tumor growth and metastasis, which was accompanied by higher percentages of activated NK cells, and higher cytotoxic activity [47]. Conversely, there are studies that show an opposite function of IL-33. One study shows that IL-33 promoted the proliferation and increased the cytotoxicity of NK cells against B16 melanoma cells [18]. It is likely that these discrepancies are due to the level of IL-33 and the primary target cells of IL-33 in these experiments are accountable for the different *in vivo* and *in vitro* effects. Thus, the immune phenotype of IL-33 is likely shaped by dose, the local environment, and the target cells. A variety of mechanisms have been previously documented to enable tumors to evade NK cell-mediated surveillance (review by Cerwenka and Lanier [48]). For example, IL-33 activates NK cells which could direct kill tumor cells, and could also induce the release of anti-tumor cytokines, such as IFN-γ and TNF-α [49, 50]. The effect of IL-33 on NK cells was also demonstrated by reduced presence of IFN-γ-expressing NK cells and increased NK cells that expressed IL-10 in tumor-bearing mice [24]. The results described in our study suggest that at least during *in vivo* tumorigenesis, IL-33/ST2 signals affect NK cell accumulation and impair NK cytotoxicity, probably by regulating NKp46 expression, which is described as essential for NK cells activation, cytokine production and release of cytolytic granules [51]. Different mechanisms have been proposed to mediate the pro-tumor or anti-tumor activity of IL-33, and further studies will be needed to determine exact effects of IL-33 and whether these findings can be generalised to different types of tumors.

In summary, our findings demonstrate that ST2-deficient mice are less susceptible to developing SCC in the skin. The IL-33/ST2 pathway contributes to SCC development by increasing the recruitment of CD4^+^ T cells, dendritic cells, and macrophages and by impairing NK cytotoxic activity. Therefore, our results confer preliminary new insights into the influence of IL-33/ST2 signaling in SCC progression and support the idea that IL-33 may be an important target for neoadjuvant treatments for cancer. However, further investigation should be performed to better understand the physiopathology of IL-33 during tumor development.

## MATERIALS AND METHODS

### Mice

Female BALB/c mice deficient in the IL-33 receptor ST2 (Il1rl1−/−, ST2KO) were provided by the School of Medicine of Ribeirão Preto, University of São Paulo. Age-matched BALB/c (wild type, WT) mice (6 to 8 weeks old) were used for all experiments. The care of the experimental animals was performed in accordance with institutional guidelines. All animal experiments were approved by the Animal Research Ethics Committee of the Bauru School of Dentistry, University of São Paulo.

### Skin carcinogenesis

The protocol for inducing two-stage carcinogenesis was previously described [19]. Briefly, the mice had their dorsal skin shaved and were topically treated with DMBA (7,12-dimethylbenz[a]anthracene, 125μg in 0,2mL of acetone). After 1 week, mice were treated with PMA (Phorbol 12-myristate 13-acetate, 10μg in 0,1mL of acetone) three times a week for 18 weeks. Mice were examined weekly. Survival rates were determined in independent groups of animals. Tumors were collected eighteen weeks after initiation.

### Histopathologic analysis

For histological analysis, formalin-fixed tumor sections were processed and embedded in paraffin using standard techniques. Longitudinal sections (5 μm-thick) were stained with hematoxylin and eosin (H&E) and analyzed by two pathologists who were blinded to the experimental groups. All sections were analyzed under an optical microscope, and microphotographs were collected using a digital camera (Leica DFC310 FX, Leica Microsystems GmbH, Wetzlar, Germany). Dysplasia scores were assigned as follows: normal (within normal limits), mild (minimal dysplastic cellular alterations were detected), moderate (moderate intensity of dysplastic features), and severe (intense presence of keratinocytes with markedly dysplastic characteristics) [52]. SCC *in situ* was distinguished from severe dysplasia by the identification of an intense presence of parakeratosis that was accompanied by the loss of the granular cell layer [53]. The following severity scores were used to score inflammation: 0 = normal (within normal limits), 1 = discrete (small, focal, or less than one-third of the region contains inflammatory cells), 2 = moderate (multifocal or locally extensive, or between one-third and two-thirds contains immune cells), and 3 = severe (more than two-thirds of the region contains immune cells) [54].

### Isolation of leukocytes

The protocol used to isolate leukocytes from the tumor microenvironment has been previously described [19]. The tissue homogenates were filtered using a 30-μm cell strainer (Falcon; BD Biosciences), the isolated cells were prepared for flow cytometry analysis, and the supernatants of the tumor samples were stored in protease inhibitor solution (Roche) at −20°C until used to measure cytokine levels. NK cells were isolated from spleens using a mouse NK Cell Enrichment Set-DM BD IMag according to the manufacturer's instructions (BD Bioscience).

### Antibodies and flow cytometry analysis

For immunostaining, FcγRs were blocked in mAb 2.4G2, and the cells were then stained for surface markers using PerCP-, PE- and FITC-conjugated antibodies against CD45 (30-F11), CD19 (1D3), CD3 (145-2C11), CD4 (RM4-5), CD8 (53-6.7), CD11b (M1/70), CD27 (LG3A10), DC (33D1), CD124 (mIL4R-M1), NKp46 (29A1.4), KLRG1 (2F1) (BD Biosciences), F4/80 (BM8) (eBioscience), and CD119 (REA189) (Miltenyi Biotec). The corresponding goat and rat isotype controls were used for each Ab that was analyzed (BD Biosciences). The data were collected using a FACSCalibur™ flow cytometer (BD Immunocytometry Systems, Franklin Lakes, NJ) and analyzed using CellQuest software (BD Biosciences).

### Cytotoxicity assay

Flow cytometric assays were used to measure *in vitro* NK activity, as previously described [55]. The mouse T-lymphoma cells YAC-1 (ATCC TIB-160) was kind gifts from Professor Thereza L. Kipnis (Biomedical Sciences Institute, University of São Paulo). YAC-1 cells (target cells) were grown in suspension in RPMI 1640 medium supplemented with 10% fetal bovine serum. Target cells were labeled with 5 μM carboxyfluorescein succinimidyl ester (CFSE) (Molecular Probes, Eugene, OR). Effector cells (splenic NK) were incubated in solutions containing various YAC-1: target (E: T) ratios in 96-well round-bottom plates for 4 hours at 37°C and 5% CO2. After incubation in culture, the cells were incubated in propidium iodide for 10 minutes and analyzed using flow cytometry. Cytotoxicity (%) was calculated using the following formula: [experimental cell death – spontaneous cell death) / (spontaneous cell death)] × 100.

### ELISA

The concentrations of IFN-γ, TNF-α, IL-12p70, IL-4, IL-13, IL-10, TGF-β, IL-17, and IL-33 were measured in the tumor supernatants recovered during tumor processing, as described in “Isolation of leukocytes” using mouse ELISA kits (BD Bioscience) according to the manufacturer's instructions.

### Statistical analysis

A two-tailed nonparametric test (Mann-Whitney *U*-test) was used for statistical comparisons of two groups, and a P value of less than 0.05, 0.01 or 0.001 was considered statistically significant.

## SUPPLEMENTARY MATERIALS FIGURES AND TABLES


