# Supplementary material for: ST2/IL-33 signaling promotes malignant development of experimental squamous cell carcinoma by decreasing NK cells cytotoxicity and modulating the intratumoral cell infiltrate
Source: Oncotarget. 2018 Jul 20;9(56):30894–904. doi: 10.18632/oncotarget.25768 (PMC6089399; doi:10.18632/oncotarget.25768)
Supplement: Supplementary file 1 [file oncotarget-09-30894-s001.pdf]

ST2/IL-33 signaling promotes malignant development of experimental squamous cell carcinoma by decreasing NK cells cytotoxicity and modulating the intratumoral cell infiltrate

SUPPLEMENTARY MATERIALS

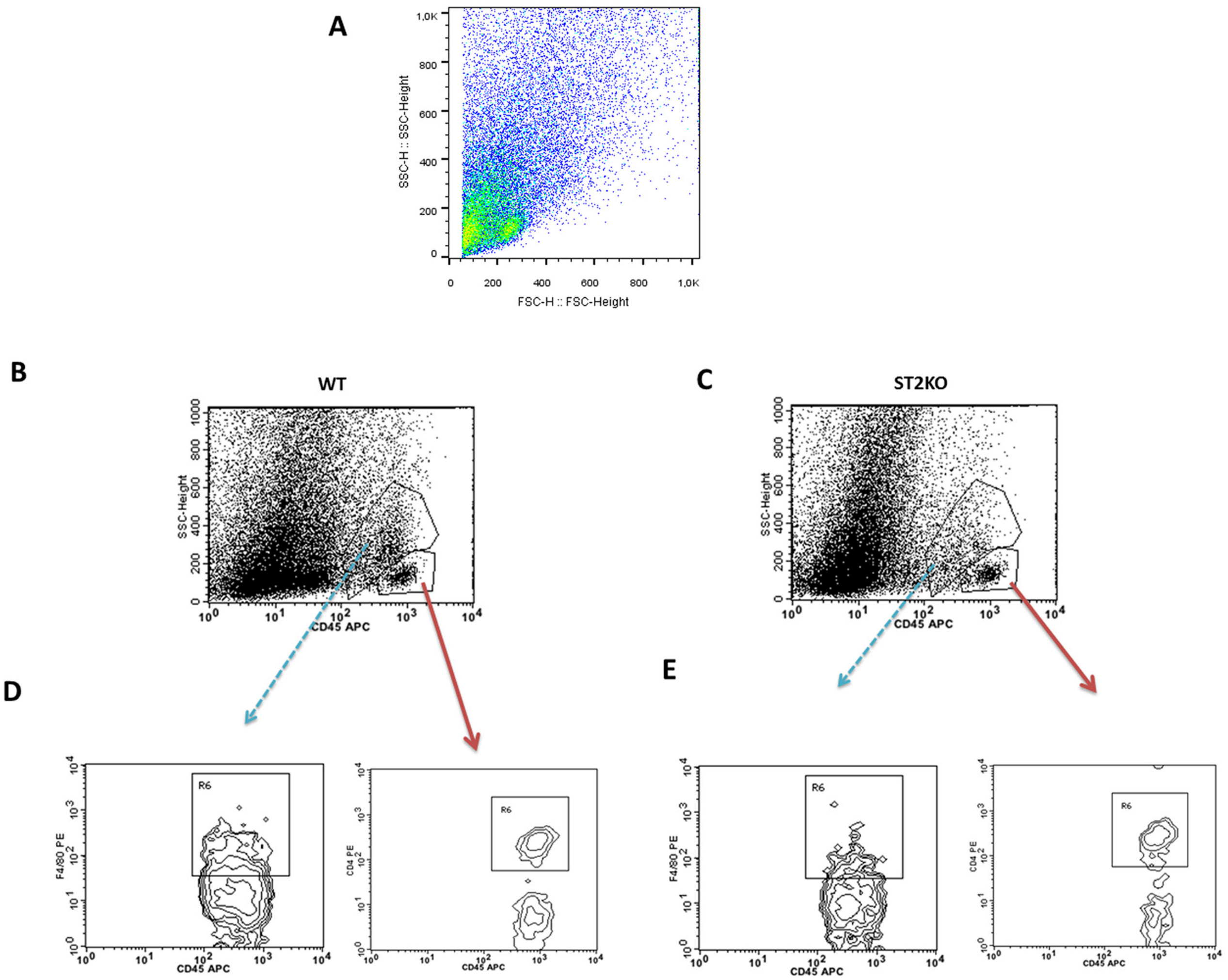

Supplementary Figure 1: General gating strategy: All events on FSC vs SSC dot plot **(A)**. Dot plots show the FACS gating strategy to distinguish macrophages (R2) and lymphocytes (R1) events between CD45+ cells from WT mice **(B)** and ST2KO mice **(C)**. Gate on macrophages (F4/80+ cells) and CD4+ T-cells from WT mice **(D)** and ST2KO mice **(E)**.
